# Identifying Borderline Ovarian Tumor Recurrence Using Routine Ultrasound Follow-Up

**DOI:** 10.3390/cancers15010073

**Published:** 2022-12-22

**Authors:** Caitlin Lazurko, Tomer Feigenberg, Joan Murphy, Kate Pulman, Genevieve Lennox, Valerie Dube, Tiffany Zigras

**Affiliations:** 1 Temerty Faculty of Medicine, University of Toronto, Toronto, ON M5S 1A8, Canada; 2 Division of Gynecologic Oncology, Trillium Health Partners, Credit Valley Hospital, Mississauga, ON L5M 2N1, Canada; 3 Department of Obstetrics & Gynaecology, University of Toronto, Toronto, ON M5G 1E2, Canada; 4 Department of Pathology, Trillium Health Partners, Credit Valley Hospital, Mississauga, ON L5M 2N1, Canada

**Keywords:** borderline ovarian tumor, ultrasound, recurrence

## Abstract

**Simple Summary:**

Borderline ovarian tumors have a favorable prognosis, and with one third of tumors diagnosed in women under 40 years of age, fertility preserving approaches are necessary. However, recurrence occurs and more frequently in those with fertility preserving treatment, but there is limited evidence regarding recurrence monitoring. The aim of this retrospective chart review was to determine if regular ultrasound follow-up is effective for recurrence monitoring in those with borderline ovarian tumors. We identified 56 patients with borderline ovarian tumors at our institution, all of whom had at least yearly ultrasound follow-up. Recurrence occurred in 6 patients and ultrasound first identified recurrence in 5 patients (83.3%), prior to findings on physical exam or patient-reported symptoms. Thus, our study suggest ultrasound as an accessible, inexpensive, and safe modality of recurrence monitoring that allows early detection of tumor recurrence and thus earlier intervention and prevention of further disease progression.

**Abstract:**

Borderline ovarian tumors (BOTs) are non-invasive tumors frequently diagnosed in young patients. Surgical removal of the uterus, fallopian tubes, ovaries, and omentum is considered definitive management, however fertility-sparing approach is a recognized option. Surveillance is important due to known recurrence, but there is controversy over the effectiveness of follow-up modalities. The objective is to determine the efficacy of ultrasound screening in identifying tumor recurrence. This retrospective chart review evaluated all patients consulted and/or treated surgically at our institution from January 2015 to June 2020 diagnosed with BOT. Patients were excluded if concurrently diagnosed with another gynecologic malignancy, did not have yearly ultrasound follow-up, or were lost to follow-up. This study included 56 patients, 17 of whom underwent fertility preserving surgery. The overall rate of recurrence was 10.7%; with recurrence rates of 23.5% for the fertility preserving surgery population and 5.1% for the definitive surgery population. Ultrasound first identified 5 of the 6 (83.3%) recurrences. Overall time to recurrence was 51.5 months. In conclusion, recurrences were identified on routine ultrasound screening prior to symptom onset or detection via physical exam in 83.3% of cases. While the best modality of follow-up remains controversial, this review provides evidence supporting the use of routine ultrasound follow-up for early detection of BOT recurrence.

## 1. Introduction

Borderline ovarian tumors (BOTs) are low-grade epithelial neoplasms with a generally favorable prognosis defined as having heterogenous epithelial proliferation and cellular atypia, while not demonstrating invasion [1,2,3,4]. BOTs are diagnosed in individuals of all ages, however there is a high prevalence in young patients, with one third of cases affecting patients under 40 [1,2,3,4,5,6,7,8]. As such, preservation of ovarian function and fertility should be considered.

The definitive management of BOTs is complete surgical resection including hysterectomy, bilateral salpingo-oophorectomy, omentectomy, and pelvic washings [9]. Given the young patient population who may wish to preserve ovarian function, surgical options to preserve fertility and/or hormonal function include unilateral salpingo-oophorectomy with or without contralateral cystectomy, unilateral or bilateral cystectomy, or bilateral salpingo-oophorectomy where the uterus is preserved in the case of prior oocyte/embryo preservation [8,9]. Regular follow-up is important as the risk of recurrence ranges from 5–30%, depending on tumor characteristics and treatment modality [3,4,5,7,8,10,11,12,13,14]. Patients undergoing fertility-sparing treatment are at a significantly higher risk of recurrence compared to those undergoing definitive management [6,11,15,16]. There is no generally accepted best practice protocols for follow-up, though physical exam, serum tumor markers such as CEA, CA19.9, and CA 125, and imaging modalities including ultrasound, CT, and PET scan are commonly used and have been shown to be effective especially when used in combination [3,8,9,17,18,19]. At our institution, standardized surveillance for all patients after either complete surgical resection or fertility-sparing surgery includes routine physical exam and regular abdominal and pelvic ultrasound every 6–12 months.

Therefore, as BOT recurrence is significant, effective surveillance protocols are important regardless of the patient’s surgical status. Early identification of recurrence allows for early intervention and prevention of further disease progression. The objective of this study is to determine if ultrasound can be used as follow-up modality for early identification of borderline ovarian tumor recurrence.

## 2. Materials and Methods

### 2.1. Patient Selection

This single-institution retrospective review was first approved by our institutional review board (#1018). All female patients seen in consultation and/or treated surgically at our institution from January 2015 to June 2020 diagnosed with borderline ovarian tumors were reviewed. All pathology was reviewed by pathologists with experience in gynecologic pathology and if surgery was completed at an outside institution, formal pathology review was required. All histology types of borderline ovarian tumor were included. Ultrasound must have been included as part of surveillance with at least one ultrasound performed per year. Exclusion criteria include patients with another gynecologic malignancy identified at the time of their surgery, concurrent malignancy diagnosis, patients without ongoing ultrasound follow-up in the last 1 year, or those lost to follow-up.

Patient demographics including age at diagnosis, BMI, gravity and parity, past medical history, tumor marker levels (CA 125, CEA, CA19.9), use of hormone therapy, colonoscopy status, tumor histology, tumor FIGO stage [20], operation, operation date, estrogen and progesterone receptor status, ultrasound dates and findings, date and mode of recurrence identification, and date of reoperation were extracted from the patient records.

### 2.2. Outcome Measure

The primary objective of this study was to determine the rate of recurrence identified by ultrasound in patients following initial surgery for BOT. Time to recurrence was defined as the number of months from initial surgery to surgery that histologically confirmed the recurrence. Progression-free survival was defined as time from initial surgery to time of surgery which confirmed the recurrence. Follow-up time was measured from date of first surgical diagnosis to date last follow-up with their gynecologic oncologist or 30 June 2020, whichever was first.

### 2.3. Statistical Analysis

Descriptive data including patient demographics, tumor histology, and treatment modalities are reported in Table 1. The Kaplan–Meier progression free survival curve were generated using Excel. *p*-values were calculated using student-*t* tests, with *p* < 0.05 considered significant.

## 3. Results

Between January 2015 and June 2020, we identified 155 patients at our institution diagnosed with borderline ovarian tumors. We excluded 76 patients due to lack of ultrasound follow-up, 18 due concurrent malignancy other than borderline ovarian tumor, and 5 who were outside the study timeline. Thus, 56 patients were included in this study. These patients were all seen by a gynecologic oncologist in person or via telehealth exam at least once per year and had yearly ultrasound screening.

Reports from the patient’s abdominal and pelvic ultrasounds were reviewed and ultrasound screening was considered significant if there was a complicated cystic mass, echogenic nodule, solid mass, mass with blood flow or if the report indicated a neoplastic mass or one concerning for tumor recurrence or neoplasm. Follicles, fibroids, and simple or likely benign cysts (no solid or nodular components, septations, or vascularization) were considered not significant. Some ultrasound findings of concern or those of undetermined significance were followed-up MRI or CT scan, as per the radiologist’s suggestion or gynecologic oncologist’s decision. One patient with increased ascites and potential nodularity in the omentum and abdomen on ultrasound and CT underwent surgical assessment which demonstrated benign multicystic mesothelium. Since this patient did not experience tumour recurrence, they were not included in the recurrence population.

Overall, 56 patients were included in this study and the patient characteristics are included in Table 1. The median age at diagnosis was 45 years (range 17–73 yrs) and the median BMI was 26.7 (range 16.4–52.0). Of the included participants, 20 (35.7%) were nulliparous and 47 were FIGO stage I (71.2%). The majority of patients had serous histology (*n* = 33, 58.9%), and 14 (25.0%) had microinvasive features identified on final pathology. Fertility preserving surgery was performed in 17 (23.5%) participants. The type of surgical procedure that each patient underwent is included in Table 2.

Recurrence was detected in 6 (10.7%) patients; the fertility preserving surgery population had a recurrence rate of 23.5% compared to 5.1% for the definitive surgery population. The overall recurrence rate as well as recurrence rates of specific populations are included in Table 3. Ultrasound first identified 5 of the 6 recurrences prior to any patient symptoms or findings on physical exam. Two patients (33%) had normal physical exams including pelvic exams during the appointment after which abnormalities were first identified on ultrasound, while one patient (17%) had a normal abdominal exam, and 3 (50%) refused physical exams. The one recurrence that was not first identified by ultrasound first presented as patient discomfort, with no abnormalities on physical exam. All patients who experienced recurrence had serous histology in our cohort. One patient had progression to low-grade serous ovarian cancer while the remainder of recurrences were of borderline histology. Follow up ultrasound identified one patient who was suspected to have recurrence, but on final pathology after surgery, the patient was diagnosed with benign Multics mesothelium representing a false positive. Of the 17 patients who underwent fertility preserving surgery, 4 (23.5%) had a recurrence detected in the ovary/ adnexa on ultrasound and all were confirmed on final pathology to be arising from the ovary. Of those who had a complete surgical resection, 2 of 39 (5.1%) patients had a recurrence. These recurrences were identified in the omentum (*n* = 1) and abdominal peritoneum (*n* = 1). Interestingly, 14 patients (25.0%) had microinvasion, but only 1 (7.1%) patient with microinvasion experienced recurrence. The recurrence rate of those with microinvasion was 7.1% which was less than those without microinvasion at 11.9%.

**Figure 1:**
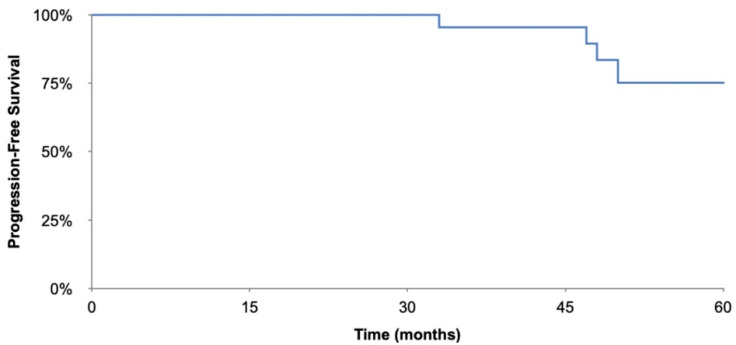
Kaplan–Meier progression-free survival curve.

Overall, the average time to recurrence was 51.5 months. Table 4 shows the overall time to recurrence as well as time to recurrence for the subgroups analysed. For the fertility preserving surgery group time to recurrence was 52.7 months versus 49.0 months in the non-fertility preserving group, Table 4. The overall survival was 100% in this study and 5-year progression free survival is 75.2%, the Kaplan–Meier curve is included in Figure 1.

## 4. Discussion

Borderline ovarian tumors (BOTs) are epithelial ovarian cancers of low malignant potential that are typically managed surgically. The definitive treatment of these tumors is complete surgical resection including hysterectomy, bilateral salpingo-oophorectomy, excision of visible tumor, omentectomy, and washings. Recurrence rates remain high, with the literature reporting rates between 5–30%, depending on the characteristics of the tumors [3,4,5,7,8,10,11,12,13,14]. These characteristics including FIGO stage, age at diagnosis, type of surgery, residual disease, implant type, microinvasion and micropapillary infrastructure [3,4,5,7,8,10,11,12,13,14]. The recurrence rate documented in the literature is consistent with the recurrence rate in our study population of 10.7%.

The demographic of patients with BOTs is such that fertility-sparing approaches must be considered, given the young age at diagnosis. However, fertility-sparing surgery confers a higher risk of recurrence, with reports ranging from 11.5–21.3% [8,13,15,16,21]. Similarly, our study identified a higher rate of recurrence in fertility sparing group compared to those receiving definitive management, 23.5% vs. 5.1%, respectively.

Regardless of the surgical approach, the optimal method of follow-up remains uncertain [3,7,11]. Many follow-up modalities are currently used including physical exam, tumor markers such as CA 125, CEA, and CA19.9, and imaging including CT, MRI, and ultrasound [6,8,9,18]. Zanetta et al. determined that ultrasound was the most effective follow-up modality compared to physical exam and blood CA 125 level [6]. Uzan et al. also determined that ultrasound most frequently identified non-invasive recurrences while CA 125 levels most frequently identified invasive recurrences [18]. NCCN guidelines suggest physical exam, imaging, and tumor marker monitoring for those who underwent completion surgery, but only suggests ultrasound surveillance for patients who have undergone fertility-sparing surgery [9].

In this study regular ultrasound monitoring identified BOT recurrence in 5 of 6 (83%) cases prior to identification by physical exam or patient symptoms regardless of the surgery that was performed, fertility preserving surgery or complete surgical resection. Our study suggests that the reliability of ultrasound as an effective modality to monitor patients for early BOT recurrence even in the absence of physical examination is reassuring. Given the small patient population, further evidence is needed to distinguish the benefit of ultrasound monitoring for patients who have undergone complete surgical resection given the lower rate of recurrence compared to fertility-sparing surgery.

The use of ultrasound as a monitoring strategy can also be safely used where there are geographical constraints for patients. With the growing acceptance of virtual care, patients can safely undergo ultrasound surveillance closer to home and have virtual follow up with their gynecologic oncologist. Fortunately, ultrasound monitoring has been shown to be a safe, accurate and cost-effective modality which can be used by patients to monitor for disease recurrence [6,18,22,23,24].

This study was not designed to assess outcomes after second surgery though it is generally accepted that early detection of recurrence is beneficial. A possible negative consequence of frequent imaging includes identification of benign lesions such as functional ovarian cysts that might be concerning for recurrence and may result in increased imaging, distress for patients and/or surgical intervention. In our study, one patient with abnormal ultrasound findings had undergone surgery for suspected recurrent disease was found to have benign multicystic mesothelioma. No other patients in our study cohort were taken to surgery for false positive findings, likely due to the small sample size, but this is a recognized possible outcome of routine ultrasound surveillance.

While the results from this study and the literature support the use of ultrasound for recurrence monitoring, it may be more effective to monitor the patient with several modalities including imaging, physical exam, or blood tumor markers to ensure recurrence is detected early. The exact time interval to monitor patients is not clearly elucidated from our data. As borderline tumors are generally slow growing, it may be reasonable to monitor every 6 months for the first 3–4 years then annually thereafter. The time period to end surveillance is also not clear in the literature, and as median time to recurrence in our study population was 51.5 months, therefore we recommend at least 5 years of surveillance. Silva et al. demonstrated that in their study population who experienced BOT recurrence, 77% occurred in the first 5 years and 34% occurred 10 years after initial diagnosis [13]. More long-term data is needed to understand when surveillance can safely be stopped [6,13].

One of the strengths of this study was that most participants were followed regularly with ultrasound surveillance at our institution and thus were followed by sonographers using the same protocol. In addition, these patients were regularly seen by a gynecologic oncologist for follow-up. A weakness of this study is that it is retrospective in nature. In addition, the sample size and the number of participants experiencing recurrence in this study was small. Another weakness was due to regular patient monitoring with ultrasound, many physical exams were deferred, typically by patient choice or due to telehealth follow up during the COVID 19 pandemic, which may have missed some signs of recurrence on physical exam. Last, tumor marker levels were not regularly monitored post-operatively which limited our ability to compare ultrasound follow-up to changes in tumor marker levels.

## 5. Conclusions

In conclusion, while borderline ovarian tumors are indolent, slow growing tumors, there is a risk of recurrence and in some cases this recurrence can be a low grade serous ovarian cancer, as such it is important that ongoing surveillance is conducted and optimized. This retrospective study, though limited by sample size, demonstrates evidence supporting the use of routine ultrasound screening for patients diagnosed with borderline ovarian tumors. We demonstrated that in patients who experienced recurrence, the majority (83.3%) were identified by ultrasound prior to patient symptoms or signs on physical exam. Routine ultrasound is therefore important in the fertility-sparing surgical population given the higher rate of tumor recurrence. While this study demonstrates that ultrasound identifies recurrence early in both the complete surgical resection and fertility-sparing populations, further evidence is needed to assess the benefit of regular ultrasounds monitoring following complete surgical resection given the lower rate of recurrence.

## Figures and Tables

**Table 1:** Baseline characteristics.

Variable	No Recurrence (*n* = 50)	Recurrence (*n* = 6)	Total (*n* = 56)	*p*
	*n*	*n*	*n* (%)	
Age at diagnosis				0.14
Median	46	37.5	45	
Range	17–73	24–52	17–73	
BMI				0.88
Median	26.5	26.4	26.7	
Range	16.4–52.0	18.6–43.3	16.4–52.0	
Nulliparous				-
Yes	16	4	20 (35.7)	
No	33	2	35 (62.5)	
Unknown	1	0	1 (1.8)	
Histology Type				-
Serous	27	6	33 (58.9)	
Mucinous	18	0	18 (32.1)	
Mixed	5	0	6 (8.9)	
Microinvasion				-
Yes	13	1	14 (25.0)	
No	37	5	42 (75.0)	
FIGO Stage				-
I	38	3	41 (73.2)	
I	2	1	3	
IA	21		23	
IB	3	2	5	
IC	12		16	
II	2		2 (3.6)	
IIA	1		1	
IIB	1		1	
IIC				
III	3		3 (5.4)	
IIIA	1		1	
IIIB	2		2	
Not available	7	3	10 (17.8)	
Location of recurrence	-			-
Ovary		4	4 (7.1)	
Omentum		1	1 (1.8)	
Peritoneum		1	1 (1.8)	
Histology of recurrence	-			-
BOT		5	5 (8.9)	
Low-grade serous		1	1 (1.8)	
Recurrence detected by ultrasound	-			-
Yes		5	5 (8.9)	
No		1	1 (1.8)	
Fertility preserving surgery				-
Yes	13	4	17 (30.4)	
No	37	2	39 (69.6)	
Length of follow-up				0.006
Average	27.3	69.8	31.9	
Range	2–71	32–104	2–104	

**Table 2:** Type of surgery each patient underwent.

Surgery		Fertility Preserving		
		Yes (*n*,%)	No (*n*,%)	Total (*n*,%)
BOT recurrence	Unilateral cystectomy	3 (17.6)	0 (0)	3 (5.4)
	Bilateral salpingo-oophorectomy, omentectomy	0 (0)	1 (2.6) *	1 (1.8)
	Bilateral salpingo-oophorectomy, hysterectomy, omentectomy	0 (0)	1 (2.6)	1 (1.8)
	Unilateral salpingo-oophorectomy, appendectomy, cystectomy	1 (5.8)	0 (0)	1 (1.8)
NoBOT recurrence	Total hysterectomy, bilateral salpingo-oophorectomy, omentectomy	0 (0)	23 (58.9)	23 (41.1)
	Unilateral salpingo-oophorectomy, omentectomy	8 (47.0)	1 (2.6) *	9 (16.1)
	Total hysterectomy, bilateral salpingo-oophorectomy, omentectomy, appendectomy	0 (0)	4 (10.2)	4 (7.1)
	Bilateral salpingo-oophorectomy, omentectomy	0 (0)	3 (7.7) *^,+^	3 (5.4)
	Unilateral salpingo-oophorectomy	2 (11.8)	1 (2.6)	3 (5.4)
	Total hysterectomy, bilateral salpingo-oophorectomy, omentectomy, appendectomy, pelvic perintonectomy	0 (0)	1 (2.6)	1 (1.8)
	Total hysterectomy, bilateral salpingo-oophorectomy, omentectomy, pelvic perintonectomy	0 (0)	1 (2.6)	1 (1.8)
	Unilateral salpingo-oophorectomy, unilateral salpingectomy, omentectomy	0 (0)	1 (2.6) ^+^	1 (1.8)
	Hysterectomy, unilateral salpingo-oophorectomy, omentectomy, pelvic peritonectomy, cholecystectomy	0 (0)	1 (2.6)	1 (1.8)
	Unilateral cystectomy	1 (5.8)	0 (0)	1 (1.8)
	Modified radical hysterectomy, unilateral oophorectomy, omentectomy	0 (0)	1 (2.6)	1 (2.4)
	Unilateral salpingo-oophorectomy, omentectomy, unilateral cystectomy	1 (5.8)	0 (0)	1 (1.8)
	Unilateral oophorectomy	1 (5.8)	0 (0)	1 (1.8)

* Patient had prior hysterectomy or prior hysterectomy with USO. ^+^ Patient chose to not have a hysterectomy.

**Table 3:** Rate of recurrence.

Type of Surgery and Tumor Characteristics	Rate of Recurrence *n* (%)
Overall recurrence rate	6 (10.7)
Fertility preserving surgery	
Microinvasion +	1 (6.2)
Microinvasion −	3 (18.8)
Non-fertility preserving surgery	
Microinvasion +	0 (0)
Microinvasion −	2 (5.0)

**Table 4:** Time to recurrence, with recurrence confirmed by tumor pathology.

Surgery	Average Time to Recurrence (Months)	*p*
Overall	51.5	0.77
Fertility preserving surgery	52.7	
Non-fertility preserving surgery	49.0	

## Data Availability

Data available upon request to corresponding author.
